# Comment on: ERS/ESTS/ESTRO/ESR/ESTI/EFOMP statement on management of incidental findings from low dose CT screening for lung cancer

**DOI:** 10.1183/13993003.01081-2026

**Published:** 2026-10-22

**Authors:** Sabrina H. Rossi, Oliver Byass, Robert Bakewell, Tristan Barrett, Simon Burbidge, Jon Cartledge, Jessica Kitt, Michelle Clark, Kerrie Massey, Grant D. Stewart

**Affiliations:** 1 Department of Surgery, University of Cambridge, Cambridge Biomedical Campus, Cambridge, UK; 2 CRUK Cambridge Centre, Cambridge Biomedical Campus, Cambridge, UK; 3 Humber, Coast and Vale Cancer Alliance, Hull University Teaching Hospital NHS Trust, Hull, UK; 4 Department of Radiology, University of Cambridge, Cambridge Biomedical Campus, Cambridge, UK; 5 Department of Radiology, Leeds Teaching Hospitals NHS Trust, Leeds, UK; 6 Department of Urology, Leeds Teaching Hospitals NHS Trust, Leeds, UK; 7 Humber Health Partnership, Hull University Teaching Hospital NHS Trust, Castle Hill Hospital, Cottingham, UK; 8 NHS Lung Cancer Screening Programme, West Yorkshire and Harrogate Cancer Alliance Team, Wakefield, UK

## Abstract

O’Dowd *et al.* [1] published the ERS/ESTS/ESTRO/ESR/ESTI/EFOMP (European Respiratory Society, European Society of Thoracic Surgeons, European Society for Radiation Oncology, European Society of Radiology, European Society of Thoracic Imaging, and European Federation of Organisations for Medical Physics) statement on the management of incidental findings on non-contrast thoracic low dose computed tomography (LDCT) for lung cancer screening. The kidney is partially imaged in 92% of people undergoing thoracic LDCT (median length of kidney visible 26% [2]).

## Shareable abstract

**Suggested updates to the ERS/ESTS/ESTRO/ESR/ESTI/EFOMP statement on management of incidental findings from low dose CT screening for lung cancer** https://bit.ly/4bavVIH

*To the Editor*:

O’Dowd *et al.* [1] published the ERS/ESTS/ESTRO/ESR/ESTI/EFOMP (European Respiratory Society, European Society of Thoracic Surgeons, European Society for Radiation Oncology, European Society of Radiology, European Society of Thoracic Imaging, and European Federation of Organisations for Medical Physics) statement on the management of incidental findings on non-contrast thoracic low dose computed tomography (LDCT) for lung cancer screening. The kidney is partially imaged in 92% of people undergoing thoracic LDCT (median length of kidney visible 26% [2]). Therefore, incidental renal findings are common but challenging to characterise due to incomplete imaging, low radiation dose and absence of contrast. In the National Lung Screening Trial (NLST) cited by the ERS/ESTS/ESTRO/ESR/ESTI/EFOMP statement, the prevalence of kidney cancer identified *via* thoracic LDCT was 0.26% (n=45 out of 17 309) [3]; however, please note that the ERS/ESTS/ESTRO/ESR/ESTI/EFOMP statement [1] incorrectly reports the prevalence as 0.08% (this requires an article correction). In the Yorkshire Lung Screening Trial (YLST), the prevalence of histologically proven kidney cancer detected on or because of thoracic LDCT by two rounds of screening was 0.26% (n=17 out of 6650, 95% CI 0.15–0.41%) [4, 5]. The prevalence of kidney cancer identified *via* thoracic LDCT was consistent in NLST and YLST despite differences in study design (the former consists of a retrospective analysis of prospectively acquired data in a portion of the NLST cohort, while the latter represents a prospective analysis) [3–5]. In the Yorkshire Kidney Screening Trial (YKST), individuals attending lung cancer screening underwent non-contrast abdominal computed tomography (CT) and the additional prevalence of kidney cancer, in the previously unimaged areas of the kidneys, was 0.25% (n=10 out of 4019, 95% CI 0.12–0.46%) [5, 6]. With lung cancer screening expanding globally, there is a need for clear guidance on management of renal findings. The American College of Radiology (ACR) White Paper for incidental renal lesions [7] provides widely used recommendations consistent with European Association of Urology (EAU) [8] and National Institute for Health and Care Excellence (NICE) [9] guidelines. While the ERS/ESTS/ESTRO/ESR/ESTI/EFOMP statement [1] cites the ACR White Paper [7], discrepancies exist which have been incorporated into the UK Lung Cancer Screening Quality Assurance process [10]. We propose amendments to align the ERS/ESTS/ESTRO/ESR/ESTI/EFOMP statement with existing evidence [7–9].

Soft tissue and mixed density renal masses (which may represent solid renal cancers and complex Bosniak cysts of category IIF–IV, respectively) have a significant chance of being malignant. As per the ACR White Paper [7], this encompasses any homogeneous renal lesion with unenhanced density between 21 and 69 HU, and any heterogeneous lesion regardless of density. The ERS/ESTS/ESTRO/ESR/ESTI/EFOMP statement suggests further imaging for soft tissue or mixed density renal masses >1 cm [1]. Solid renal masses <1 cm have ∼60% malignancy rate on pathology, and although unlikely to grow rapidly or metastasise at this size, some of these will be very early aggressive cancers [7, 11, 12], and this is reflected in existing guidelines. The ACR White Paper recommends further imaging for such lesions of any size, with either contrast-enhanced CT or magnetic resonance imaging (MRI) and subsequent follow-up [7]. Both ACR and EAU recommend active surveillance rather than discharge as part of patient-centred decision-making [7, 8]. NICE guidelines advise active surveillance in patients with localised solid renal masses, Bosniak III or IV cysts, that are <2 cm (or active treatment in patients who decline active surveillance) [9]. While the ACR White Paper recommends further imaging for incidental soft tissue or mixed density renal masses of any size detected on abdominal non-contrast CT, we acknowledge the inherent limitations of applying attenuation-based criteria to partially visualised renal lesions <1 cm on thoracic LDCT. It is crucial to strike a balance between early cancer detection and over-investigation (with potential over-treatment, patient anxiety and even patient harm) of incidental renal lesions <1 cm on LDCT thorax. In the absence of dedicated data on clinical outcomes of incidental renal lesions identified on lung cancer screening, we suggest a pragmatic approach, updating the ERS/ESTS/ESTRO/ESR/ESTI/EFOMP statement with the addition that soft tissue or mixed density renal masses <1 cm should be referred for further assessment with contrast-enhanced CT or MRI, or evaluated at the next screening round, based on patient preference (table 1) [7, 13]. Re-assessing these lesions <1 cm at the next screening round, at a 2-year time interval, would enable earlier detection (and potentially treatment) of those which may have aggressive potential, while mitigating over-diagnosis. The focus on patient choice drives patient-centred decision-making. Future research or emerging clinical evidence may allow further updated recommendations.

**TABLE 1:** Existing recommendations regarding investigation and management of incidental kidney findings from lung cancer screening and suggested updates

	ERS/ESTS/ESTRO/ESR/ESTI/EFOMP statement [1]	ACR White Paper [7]	Recommended update to ERS/ESTS/ESTRO/ESR/ESTI/EFOMP statement [1]
**Soft tissue or mixed density renal masses which may represent solid renal cancers and complex Bosniak IIF–IV cysts, respectively**	Soft tissue or mixed density renal mass >1 cm: refer for further assessment with contrast-enhanced CT or MRI	Soft tissue or mixed density renal mass: refer for further assessment with contrast-enhanced CT or MRI	Soft tissue or mixed density renal mass >1 cm: refer for further assessment with contrast-enhanced CT or MRI and Soft tissue or mixed density renal mass <1 cm: refer for further assessment with contrast-enhanced CT or MRI, or evaluate at the next screening round, based on patient preference
**Homogeneous hypodense or hyperdense cysts which may represent simple Bosniak I or Bosniak II cysts, respectively**	Homogeneous hypodense or hyperdense cysts <3 cm do not require further investigation: larger lesions should be evaluated in the next lung cancer screening round	Homogeneous hypodense or hyperdense cysts do not require further investigation (regardless of size)	Homogeneous hypodense or hyperdense cysts do not require further investigation or follow-up

ERS: European Respiratory Society; ESTS: European Society of Thoracic Surgeons; ESTRO: European Society for Radiation Oncology; ESR: European Society of Radiology; ESTI: European Society of Thoracic Imaging; EFOMP: European Federation of Organisations for Medical Physics; ACR: American College of Radiology; CT: computed tomography; MRI: magnetic resonance imaging.

With regard to benign cysts, the ERS/ESTS/ESTRO/ESR/ESTI/EFOMP statement suggests that homogeneous hypodense or hyperdense cysts <3 cm do not require further investigation, while larger lesions should be evaluated in the next lung cancer screening round [1]. The ACR White Paper states that on unenhanced CT a homogeneous lesion between −10 and 20 HU is very likely to be a simple cyst (Bosniak I) and a homogeneous lesion ≥70 HU is very likely to be a hyperdense Bosniak II cyst, neither of which require further investigation, regardless of size [7]. The EAU guidelines confirm that Bosniak I–II cysts require no further investigation nor follow-up [8]. Therefore, to ensure consistency with ACR and EAU, we suggest updating the ERS/ESTS/ESTRO/ESR/ESTI/EFOMP statement by removing the size criterion: homogeneous hypodense or hyperdense cysts require no further investigation or follow-up (table 1). In YLST, the prevalence of newly diagnosed benign cysts (Bosniak I–II) on thoracic LDCT performed for lung cancer screening was 0.56% (n=37 out of 6650) [6]. It is estimated that each year 325 000 people will undergo thoracic LDCT as part of lung cancer screening (first lung scan) [14]. Assuming a prevalence of 0.56%, approximately 1800 individuals will have newly diagnosed benign cysts (Bosniak I–II) per year. Although data regarding the size of these benign cysts is unknown, it may be that a proportion of these patients (*i.e.* those with cysts >3 cm) will avoid having an abdominal CT if our suggested updated guidance are adopted, with accompanying reductions in costs and participant cancer worry.

In summary, incidental renal lesions are common extrapulmonary findings on thoracic LDCT. A key challenge is that the kidneys (and any kidney lesions) are only partially visualised on thoracic LDCT, meaning some individuals require further imaging to characterise these. Given kidney cancers are often asymptomatic until advanced, detecting renal tumours during lung cancer screening offers valuable opportunities for earlier diagnosis. We advocate that renal masses detected incidentally on thoracic LDCT should be investigated as per ACR, EAU and NICE guidelines and suggest updates to the ERS/ESTS/ESTRO/ESR/ESTI/EFOMP statement, while those with benign cysts can be reassured without further renal imaging or follow-up.
